# Updating the seismotectonic setting for the Gulf of Aqaba

**DOI:** 10.1038/s41598-023-38759-6

**Published:** 2023-07-19

**Authors:** Mona Abdelazim, Mohamed N. ElGabry, Mohamed M. Gobashy, Mohamed H. Khalil, Hesham M. Hussein

**Affiliations:** 1 Egyptian National Data Center (ENDC), National Research Institute of Astronomy and Geophysics (NRIAG), Helwan, Egypt; 2 Geophysics Department, Faculty of Science, Cairo University, Giza, Egypt

**Keywords:** Solid Earth sciences, Seismology, Tectonics

## Abstract

The Gulf of Aqaba is known for its high seismic activity in Egypt and the Middle East. An inversion technique was applied to 113 earthquakes of magnitude 2.5 to 7.2 to distinct subsets of data based on tectonic regionalization to define the stress regime in the Gulf of Aqaba involving the Eilat basin, Aragonese basin, and Dakar basin. The stress inversion revealed two active stress patterns; an active strike-slip in the Eilat basin and a dominant extensional regime in the Dakar basin, whereas both strike-slip and extensional regimes coexist in the Aragonese basin. The stress pattern in the Eilat basin is consistent with the movement along the Dead Sea Transform Fault. In contrast, the extensional regime in the Dakar basin aligns with the extensional stress field throughout the northern Red Sea. The coexistence of two dominant types of stress regimes in the Aragonese basin is likely a result of the superposition of the two main neighbouring stress regimes: the strike-slip regime along the Gulf of Aqaba Dead Sea Transform Fault and the extensional stress state across the northern Red Sea. The orientations of the minimum principal stress in the three basins are almost similar, indicating ENE trending, nearly horizontal extension.

## Introduction

**Figure 1:**
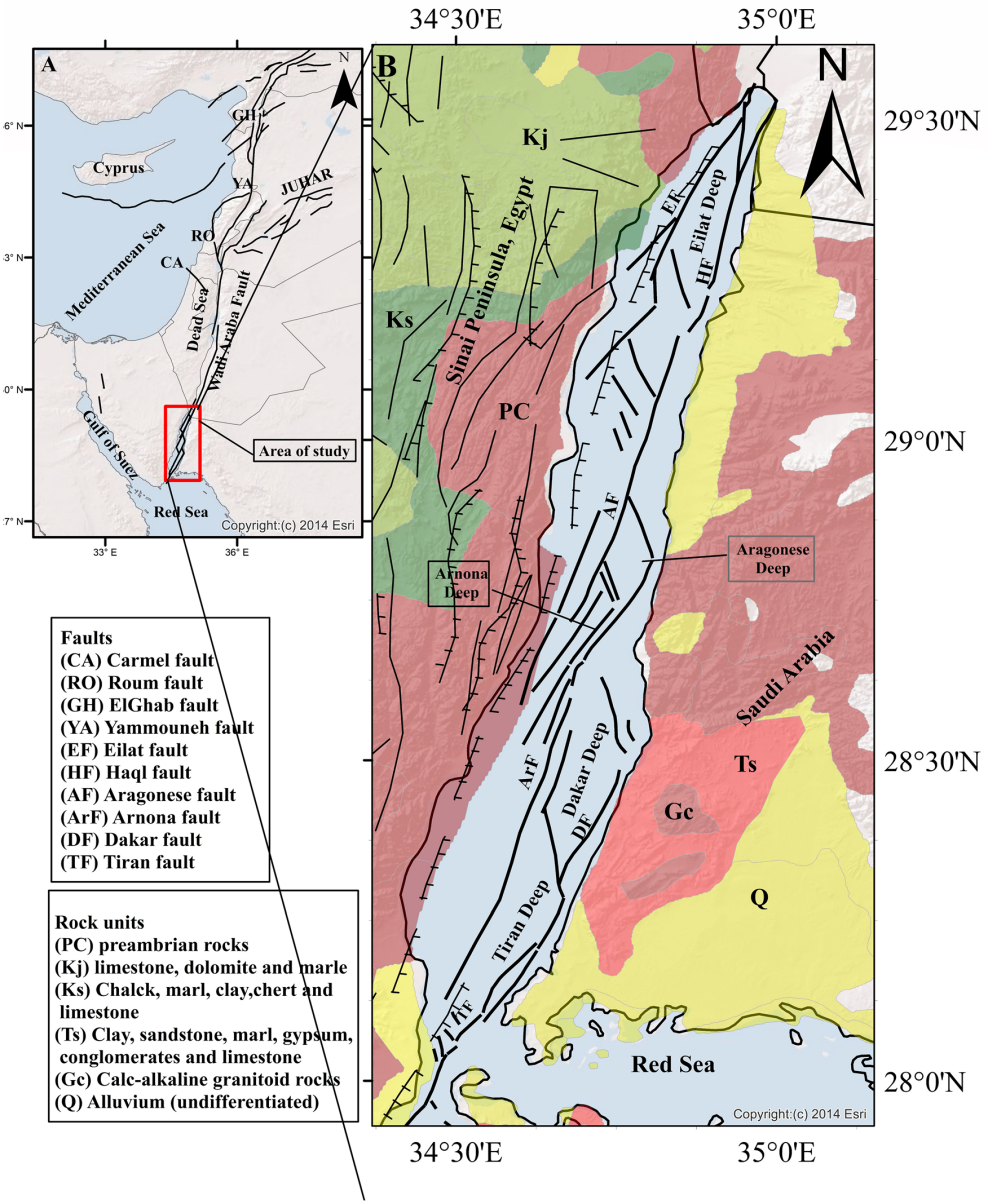
Tectonic setting of the Dead Sea Transform Fault (DSTF) and Gulf of Aqaba (the area of study) the faults from^44^ and the geological map from the Annals of the Geological Survey of Egypt. This map was created by using ArcGIS 10.3 software and basemap produced by^70^.

The Gulf of Aqaba represents the link (transition) between the Dead Sea Transform Fault (DSTF) and the Red Sea, complicating its tectonic behaviour (Fig. 1). The left lateral Strike-slip motion along the NNE-SSW trending faults in the Gulf of Aqaba is a common mode of deformation that was represented by focal mechanism solutions for the largest events in the Gulf. This type of motion is the main tectonic feature along the DSTF. However, the normal component has also strongly appeared in the Gulf of Aqaba, particularly for moderate magnitude events, which is the predominant feature of the Red Sea, particularly the northern part. The normal and oblique components in the Gulf can't be neglected, but they could be attributed to the effect of the ongoing opening of the Red Sea.

Stress is the fundamental factor controlling rock deformation. Therefore, understanding the tectonic process and the behaviour of faults in seismically active zones requires knowledge of the stress state. Studying stress is essential for civil, mining, and petroleum engineering^1^. Determining the state of stress within the Earth's crust is especially essential for geologists and geophysicists since it can improve their understanding of geodynamic processes^2^. The principal stress orientations can be determined from either fault-slip data or earthquake focal mechanisms, but the best way is to derive the stress from a set of available focal mechanism solutions from a specific region characterized by homogenous stress fields, using the stress tensor inversion technique. Focal mechanism solutions (FMS) provide essential data for many seismological phenomena, such as stress field changes in various active tectonic regions^3^, the geometry of faulting, tectonic regime assignment, maximum horizontal stress orientations, seismotectonics, and so on. Although the individual focal mechanism solutions yielded P, T, and B axis orientations which provide some general observations concerning the general tectonic activity, they deviate significantly from the principal stress direction, particularly when earthquakes occur in weak zones and on pre-existing faults^2,4^. Therefore, the stress tensor inversion approach is widely employed to accurately determine the stress state and the principal stress axes orientations which probably explain the observations from the focal mechanism solutions of earthquakes^5–8^. The maximum σ1, intermediate σ2, and minimum σ3 principal stress axes orientations, as well as the scalar that specifies the relative magnitude of the principal stresses, are obtained using this technique. Different stress inversion schemes are developed and implemented in various tectonic environments and scales^9,10^.

Stress tensor in the Gulf of Aqaba was studied and introduced by several authors such as^11–16^. These studies produced a variety of findings. Some results indicatedthat the Gulf of Aqaba only experiences a strike-slip regime, while others indicated that the Gulf is characterized only by a normal stress regime. Yet other results showed a strike-slip stress regime with the normal component. The stress tensor inversion of 14 focal mechanisms using the Gephart and Forsyth technique revealed that the Gulf of Aqaba exhibits a strike-slip regime with SHmin of 52° and SHmax of 142°^11^. The stress tensor inversion of 19 earthquake focal mechanisms in the northern part of the Gulf demonstrated a strike-slip regime with horizontal SHmin and SHmax stress axes oriented N55°E and N145°E respectively while the inversion of 6 events in the southern part indicated also strike-slip regime with different stress axes orientations. The SHmin orientation is nearly N93°E, whereas the SHmax orientation is N4°E^12^. The existence of the left lateral strike-slip movement with a normal component in the Gulf of Aqaba was demonstrated by^13^. The geologic fault slip direction investigation in the Gulf of Aqaba revealed a normal fault stress regime, in the southern part (Dakar basin), with an ENE-WSW extensional trend, and that this mode extends to the Aragonese basin^14^. The SHmin orientation is N53°E, while the SHmax orientation is N143°E. Stress tensor inversion of 35 focal mechanisms in the Gulf of Aqaba indicated a predominantly strike-slip stress field represented by SHmin of 90.5° and SHmax of 357°^15^. The stress inversion evaluation retrieved from 96 focal mechanism solutions in the Gulf of Aqaba revealed that the area is dominated by a normal stress field with a SHmax of 159°^16^.

The focal mechanism solutions in the Gulf of Aqaba are intensely studied by several authors^11–13,15,17–29^. These studies revealed a variety of fault types, including normal faults, strike-slip faults, and oblique-slip faults, reflecting the complexity of the structure in the Gulf of Aqaba, These solutions demonstrated that the main fault trends in the Gulf of Aqaba are confined to the NNE–SSW, which primarily represents the left lateral strike-slip motion that is parallel to the Gulf while the NW–SE and the WNW–ESE fault trends represent the normal dip-slip motion that is distributed along the Gulf basins margin.

This work aims to update the current focal mechanism catalogue for the Gulf of Aqaba earthquakes and to establish the current stress state relying on the stress inversion of the focal mechanism solutions, as well as to find the extent to which the effect of the stress field in the Red Sea manifests within the Gulf. Stress field investigation is of great importance for understanding the specific seismotectonics of the Gulf of Aqaba. For the current work, we chose to examine and evaluate the stress state for each basin in the Gulf (Eilat, Aragonese, and Dakar basins) in contrast to the previous studies that evaluated the stress field for the entire Gulf.

## Geology and tectonic setting

The Red Sea and the Gulf of Aqaba constitute the plate boundaries that separate Africa-Nubia, Arabia, and Sinai. The Red Sea rift system undergoes continuous oceanic spreading; the most active part is the spreading centres, particularly in the south. The Levant tectonic model links the oceanic spreading centre in the Red Sea to the westward offset of the Anatolia fault via the Gulf of Aqaba-Dead Sea transform^30–32^. According to these authors, the early Miocene tectonic opening in the Gulf of Aden and the Red Sea pushed the Arabian plate northward, resulting in a sinistral displacement of 105 km along the Levant Rift^30,33–35^ observed that the offset along the pre-existing geologic features or pinning points revealed that overall slip corresponds to 107 km, with 62 km of this slip occurring through the Early Miocene to Pliocene, followed by 45 km occurring from post-Pliocene to Recent. Africa and Arabia are recently diverging across the southern Red Sea at a rate of 1.7 ± 0.1 cm/year in a NE-SW direction^36^ while 2.4 cm/year according to^32^ where suggested that there is an acceleration in the Red Sea opening resulted at least partially from the completion of the oceanic spreading centre along the length of the Gulf of Aden, decoupling the Arabia and Somalia plates. In the northern Red Sea, the opening rate reduces to about ~ 1 cm/year^37^. The Plate reconstructions suggest that these opening rates must have been about half these values before 11 ± 2 Ma^38^. This opening led to the generation of NNW-SSE oriented Gulf of Suez continental rift (the western arm of the Red Sea), the Red Sea, and the NNE-SSW trending Gulf of Aqaba transform. Delaunay et al.^39^ Shows the main difference between the southern and northern Red Sea lies in the direction and rate of plate motion as a result of the geodetic constraints given by the Euler pole of rotation to the north. Consequently, the ca. 30° counterclockwise strike change and halving of the spreading rate (ca. 16 to ca. 10 mm. year^−1^ )between 18°N and the Suez triple junction result in a shift from slow, orthogonal to oblique.

The opening of the Red Sea and Gulf of Suez began in the late Oligocene and continued thereafter, however, the movement of the Dead Sea Transform has replaced the extension in the Suez Rift and allowed the ongoing opening of the Red Sea Basin^40^, which may have occurred at the end of Miocene simultaneously with the opening of the Gulf of Aqaba^41^. The Gulf of Aqaba represents a transition stage from the spreading zone in the Red Sea to the DSTF which is characterized by left-lateral strike-slip displacement^35,42^ was one of the first researchers to identify this fault zone and its associated sense of motion. The Gulf of Aqaba is a tectonically active region that forms an "echelon" strike-slip fault system along the plate boundary between Nubia-Sinai and Arabia. It occupies the southernmost part of the Dead Sea Transform Fault (DSTF) and also has three main pull-apart basins which developed between the overlapping ends of a major left-lateral strike-slip fault. Eilat Deep, Aragonese Deep, and Dakar Deep are the three pull-apart basins^43^. Based on multibeam bathymetry^44^, the Gulf of Aqaba can be categorized into six basins, including the Dakar Deep, Tiran Deep, Hume Deep, Aragonese Deep, Arnona Deep, and Eilat Deep Fig. 1. The two basins in the northern part of the Gulf of Aqaba, the Eilat Deep and the Aragonese Deep are well separated and exhibit a typical pull-apart morphology, according to multibeam bathymetric data. The smaller Arnona Deep is situated close to the Egyptian coast, southwest of the Aragonese Deep. Further south, the Dakar and Tiran Deeps are surrounded by a common series of faults despite being morphologically distinct and separated by just a small high. The Eilat Basin is bordered by strike-slip and normal fault systems, with the first group representing the Eilat and Aragonese faults, which border the basin from the west and east, respectively. The second group belongs to normal faults that run along the basin's northern and southern limits. The Aragonese Basin is also bordered by strike-slip and normal fault systems. Strike-slip faults include those that border the Aragonese basin from the west and the east respectively, and are known as the Aragonese and Arnona faults. The basin is surrounded on the north and south by NW-trending lines, which constitute the normal faulting. A smaller secondary basin called the Arnona Basin exists at the southern end of the Aragonese fault, to the southwest. This Basin is isolated from the Aragonese basin by an elevated sea floor, flanked by normal faults on each side and bordered on the west by oblique-slip faults. At its southern extreme, the Dakar fault, which defines the southernmost basins, the Dakar and Tiran, exhibits dip-slip normal motion. In these basins, there is no evidence of strike-slip motion. The Tiran Basin's southern boundary is marked by a set of NW-trending short parallel normal faults.

On the longer geological timescale, the DSTF extends over 1200 km from the southernmost end of the Gulf of Aqaba and links extensional tectonics in the Red Sea to contraction tectonics in the Zagros-Bitlis convergence zone of eastern Turkey^34,42,45^ where it forms the northern part of the Syrian-African rift system. The largest earthquakes in the Gulf of Aqaba region are categorized as shallow earthquakes. Their depths did not exceed 15 km, indicating that these earthquakes occurred in the Gulf of Aqaba's upper crust, which is marked by a continuous vertical transition from brittle deformation near the surface to ductile deformation at lower crustal depths^46^. El-Isa^47^ reported that the majority of small magnitude earthquakes in the Gulf of Aqaba occur in the upper crust at depths shallower than 20–22 km, with a significant majority occurring at depths of 15 km. This pattern similarly reveals a brittle upper crust and a ductile lower crust. These earthquakes do not reach the Moho because the depth down to the Moho discontinuity in the Gulf of Aqaba thins from the north (35–37 km) to the south (27–28 km), suggesting a southward increase in extension towards the Red Sea, which likely governs the structural history of the southern part of the gulf^48,49^.

Global positioning system (GPS) observations revealed that the present left lateral displacement rates along the DST fault are currently around 5 mm/year^50–53^. while geological evidence indicated faster rates of long-term displacements between 5 and 10 mm per year in the past starting from the initiation of the DST 20–15 Ma ago^34,54^.

### Methodologies of focal mechanism solutions and stress tensor inversion

We compiled all available data of earthquakes presented in this study from different sources, among which (1) the focal mechanism solutions published in various literature between 1982 and 2011; (2) polarities of P waves first onset, amplitudes of S waves and S/P amplitude ratio between 2012 and 2021^55^. The polarity and amplitude data were extracted from the digital waveforms through several local and regional agencies to increase station numbers and reduce the azimuth gap as much as possible. These data were obtained from the databases of the following sources (1) Egyptian National Seismological Network (ENSN), (2) Egyptian Strong Motion Network (ESMN), (3) International Data Center (IDC), (4) Incorporated Research Institutions for Seismology (IRIS), (5) Observatories & Research Facilities for European Seismology (ORFUES) and (6) European Mediterranean Seismological Centre (EMSC). The focal mechanism catalogue contains 113 events with 2.5 ≤ ML ≤ 7.2 which occurred between 1982 and 2021. In the current study, the new focal mechanism solutions were constructed for 9 events with 3.6 ≤ ML ≤ 4.2, covering the period from 2012 to 2021. The list of these earthquakes is shown in Table 1 and their fault plane solutions parameters in Table 2.

**Table 1:** the hypocentral location of earthquakes that happened in the Gulf of Aqaba source region from 2012 to 2021.

Event_no	Date			Time			location		Depth	mag
	Year	Month	Day	Hour	Minutes	Seconds	Lat	Long	(Km)	ML
Ev01	2015	6	27	15	34	2	28.89	34.83	20	6.13
Ev02	2015	6	28	8	27	52	28.88	34.70	20.6	4.15
Ev03	2015	6	29	7	2	35	28.89	34.75	15.8	3.81
Ev04	2015	7	8	2	15	33.48	29	34.7	18.1	4.22
Ev05	2016	5	16	1	45	85.15	28.5	34.7	6.49	5.5
Ev06	2016	8	13	3	6	14.38	28.5	34.8	19.1	3.93
Ev07	2016	11	29	17	1	14.93	28.6	34.7	19.7	4.59
Ev08	2017	05	19	14	16	21	27.94	34.563	3.55	4.1
Ev09	2020	04	05	05	04	06	29.00	34.67	15.23	4.2

**Table 2:** the focal mechanism parameters for earthquakes that occurred in the Gulf of Aqaba source region from 2012 to 2021.

Ev.no	Mechanism						P-axis		T-axis		Software name	References
	Strike1	Dip1	Rake1	Strike2	Dip2	Rake2	az	pl	az	pl		
Ev01	41	83	− 23	134	67	− 172	355	21	89	11	SU & Focmec	This study
Ev02	134	35	− 111	339	58	− 76	285	73	59	12	SU & Focmec	–
Ev03	336	31	− 122	192	64	− 72	134	66	269	18	SU & Focmec	–
Ev04	54	84	− 29	184	61	− 173	7	25	105	16	SU & Focmec	–
Ev05	142	60	− 178	51	88	− 30	3	22	101	19	SU & Focmec	–
Ev06	134	44	− 53	267	56	− 120	122	64	18	6	SU & Focmec	–
Ev07	72	77	− 37	172	54	− 164	26	35	126	15	SU & Focmec	–
Ev08	168	48	− 75	327	44	− 106	146	79	247.45	2	SU & Focmec	–
Ev09	150	64	− 46	265	50	− 145	110	50	210	8	SU & Focmec	–

The initial focal mechanism solution for the new 9 earthquakes was constructed from the first P-wave polarity using PMAN software of^56^. Subsequently, the focal mechanism solutions were recalculated using focmec software^57^. This software calculates the focal mechanism solutions depending on the polarity of the first P-wave onset, polarities of S_H_ and S_V_ phases, and the amplitude ratios of (S_H_/P)_,_ (S_V_/P), and (S_V_/S_H_). The first-motion amplitude data (S_V_/P, S_H_/P, and S_V_/S_H_) contribute to improving the identified solutions. FOCMEC conducts a grid search over all possible solutions based on the user-selected parameters, including polarity errors, the range of disagreement between the observed and calculated amplitudes, and the number of ratio errors that are permitted outside a particular range. The corresponding amplitude ratio error is calculated according to the maximum allowed log_10_ ratio^58^. Our solutions are estimated using a 5° grid search while the maximum allowable log_10_ ratio is 0.6.

**Figure 2:**
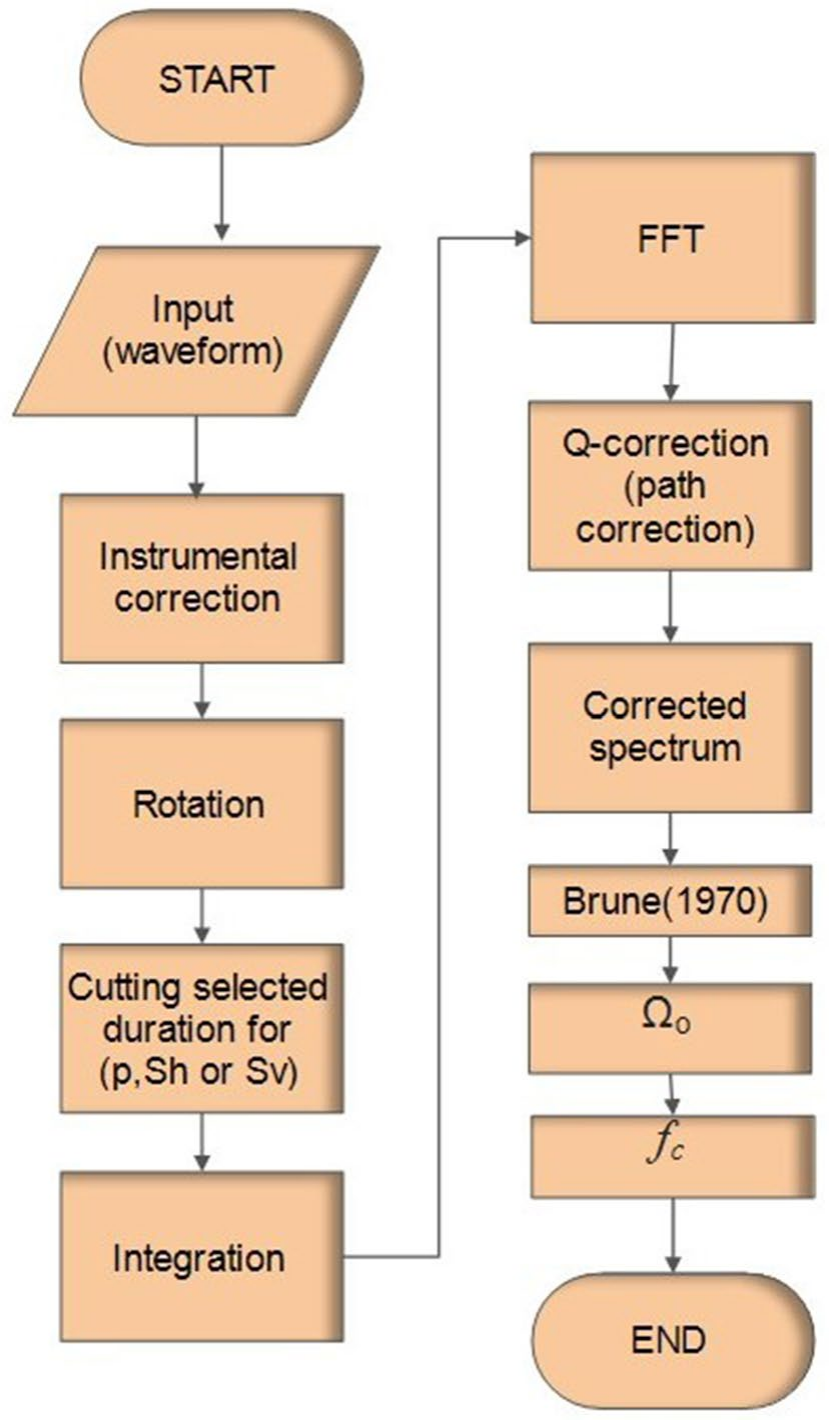
Flowchart showing sequence steps for obtaining the corrected spectrum, corner frequency (*fc*), and flat part (Ω_°_).

**Figure 3:**
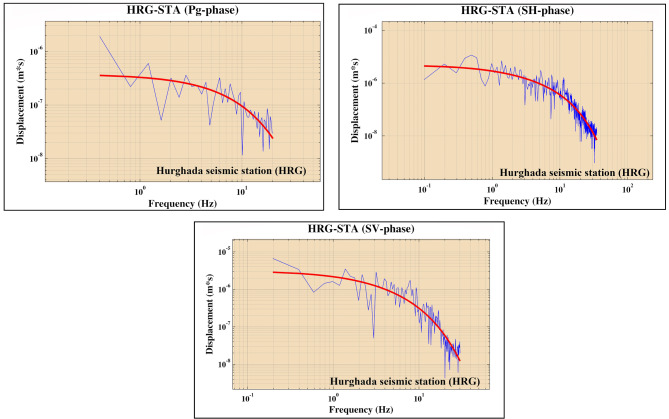
Displacement spectra for Hurghada seismic station (HRG) for different phases. The blue line shows the spectrum while the red line shows the fitted omega-square source model curve.

**Figure 4:**
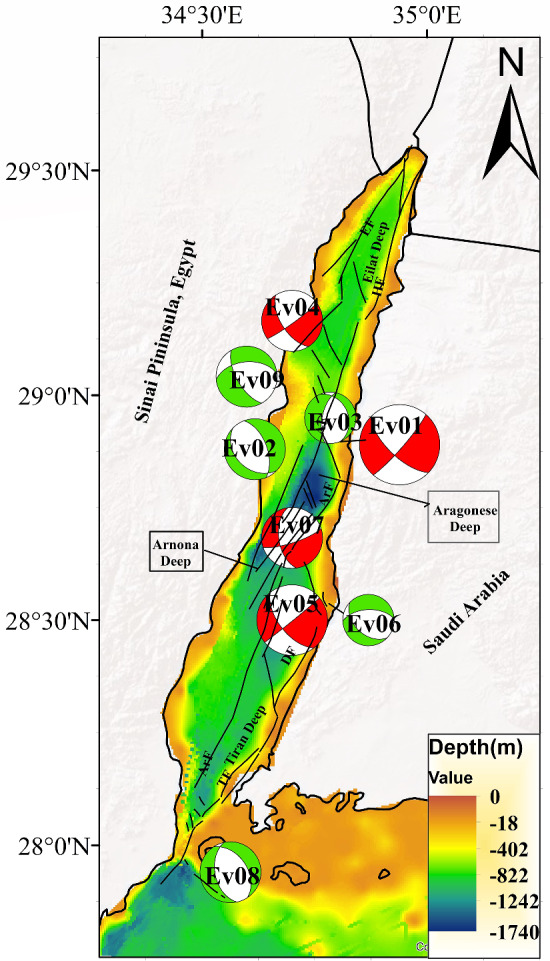
The map shows fault plane solutions for earthquakes in the Gulf of Aqaba region and the epicentres distribution of these earthquakes and we added a bathymetric map from^71^. We created this map by using ArcGIS 10.3 software.

**Figure 5:**
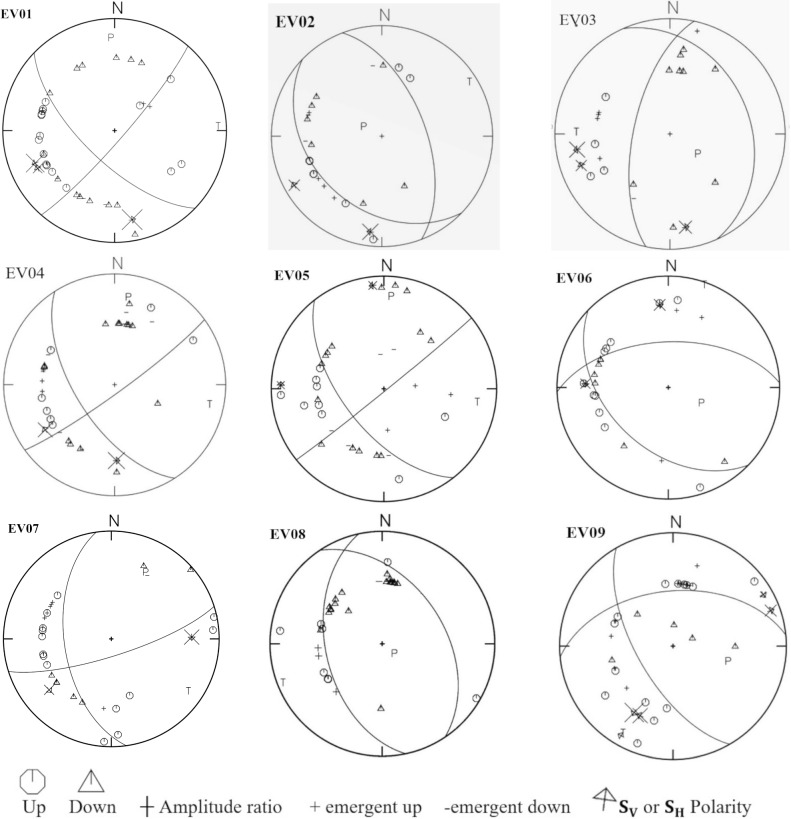
The constructed Focal mechanism solutions for earthquakes in the Gulf of Aqaba source region. These solutions were constructed depending on polarities of P, S_H_, and, S_V_ phases and spectral amplitude ratio of (S_H_/P)_,_ (S_V_/P), and (S_V_/S_H_) by FOCMEC software.

The polarities and amplitudes of the P-phase were picked from vertical components seismograms, while ${{\varvec{S}}}_{{\varvec{V}}}$ and ${{\varvec{S}}}_{{\varvec{H}}}$ polarities were picked from the radial and transverse components generated by the rotation of the two horizontal components using the seismic analysis code (SAC). Before making rotation some procedures must be applied, including (1) instrumental correction for selected three components stations (2) calculating spectrogram to detect the exact duration of different phases to avoid interference and contamination between phases as shown in Fig. S2 (supplementary material) (3) rotation of the two horizontal components to obtain radial and transverse components of S-phase. Following the rotation, various spectral analysis procedures are employed to get the corrected spectrum, as explained in Fig. 2. Finally, the corrected observed spectrum of the earthquake was fitted to the theoretical curve of the Brune source model^59^. This fitting offers the spectral amplitude of the flat part Ω◦ of the different phases*.* Once we have obtained Ω◦ of the various phases, as illustrated in Fig. 3, we then calculate different amplitude ratios such ${{\varvec{S}}}_{{\varvec{V}}}/{\varvec{P}}$, ${{\varvec{S}}}_{{\varvec{H}}}/{\varvec{P}}$ and **S**_**V**_**/S**_**H**_. Figure 4 shows the spatial distribution of the new focal mechanism solutions that we have constructed for the nine earthquakes added by this study. The focal mechanism solutions for these events are shown in Fig. 5 and the details (are shown in Fig. S1 in the supplementary material).

In addition, we computed the seismic moment and energy released for the various types of mechanisms involved in the nine earthquakes that occurred in the Gulf of Aqaba between 2012 and 2021. We divided the mechanisms into strike-slip and normal mechanisms as a result, and we estimated the total seismic moment and energy released in each basin as well as for the whole Gulf (Table 3).

**Table 3:** The seismic moment and energy released from the earthquakes in Gulf of Aqaba from 2012 to 2021.

Index	Seismic moment (dyne cm)	Energy release (J)	
Eilat basin			
Strike-slip mechanism			
Ev04	6.43948E+21	2.08E+09	
Aragonese basin			
Strike-slip mechanism			
Ev01	5.876e+23	3.17e+12	
Ev07	9.34e+22	1.62e+11	
Sum	6.81e+23	3.332e+12	
Normal mechanism			
Ev02	4.6E+21	1.23E+09	
Ev03	9.28E+20	7.212E+07	
Ev09	3.5169E+22	1.78E+10	
Sum	4.0697e+22	19.1e+9	
Dakar Basin			
Strike-slip mechanism			
Ev05	1.3426E+23	2.9751E+11	
Normal mechanism			
Ev06	4.8E+21	1.38E+09	
Ev08	6.9724E+21	2.54E+09	
Sum	1.17724e+22	3.9e+09	

Gulf of Aqaba			
Sum of all Normal mechanisms		Sum of all Strike-slip mechanisms	
Seismic moment (dyne cm)	Energy release (J)	Seismic moment (dyne cm)	Energy release (J)
5.24694e+22	23e+09	8.216995e+23	3.63159e+12

In the current study, we applied the stress tensor inversion technique of^6^ to evaluate the stress field for each basin in the Gulf to find out whether the Gulf is influenced by the Red Sea opening or if it is tectonically linked to movement along the DSTF, using the focal mechanism database constructed in this work. In contrast to our work, earlier research examined the Gulf's overall stress status.

Various approaches for performing the inversion have been proposed^5,60–62^ developed one of the most common stress inversion techniques**;** modifications and extensions were proposed by^9,60^. These methods assume that (1) the tectonic stress is uniform (homogeneous) in the region (2) earthquakes occur on pre-existing faults with varying orientations (3) These inversion methods are based on the Wallace-Bott hypothesis, which assumes that slipping (d) on a fault surface occurs in the direction of maximum shear stress (τ) and it applies to both newly formed faults^63^ and reactivated ones. The angle between (τ) and (d) is the misfit angle (α) which should be minimized for each earthquake (i). Angelier^64^ identified four independent parameters that represent the orientation of the reduced stress tensor. These parameters include σ, σ_2_, σ_3_ and the ratio of the principal stress difference R. The stress ratio, R, defines the geometry of slip-along fault planes and governs the orientation of shear stress for any particular plane^63^. In the current study, we used the stress tensor software (TENSOR software) of^6^ for identifying the four parameters of the reduced stress tensor**.** This software does not require a prior decision regarding which of the two nodal planes to use before inversion. In the initial stage of the processing, the four parameters of the reduced stress tensor are estimated roughly using the improved Right Dihedron Method, which is based on^65^ work. Additionally, this technique eliminates focal mechanisms that are incompatible with the predominant data set. The filtered focal mechanisms will be used as the first step in the Rotational Optimization inversion technique. The Rotational Optimization inversion technique employs an iterative grid-search of stress tensors to minimize the angle deviation (α) between the modelled and observed slip lines on the plane, preferring a higher shear stress magnitude ׀τ(i) ׀ and lower normal stress ׀v(i) ׀magnitude. The Tensor software employs F5 misfit function while^6^ employs F3. The Rotational Optimization inversion technique initially inverted both nodal planes; the nodal plane that best fits the uniform stress field would be chosen as the actual fault plane^61^.

The orientations of the horizontal stress axes (SHmax and Shmin) are computed with the formula of^66^. The stress regime index R′ and the stress ratio R values are used to define and identify the stress regime, as follows:

$$\begin{aligned} {\text{R}}\prime & = {\text{R}}\;{\text{for}}\;{\text{normal}}\;{\text{faulting}}\;{\text{regimes }}\left( {{\text{NF}}} \right) \\ {\text{R}}\prime & = \, \left( {{2} - {\text{R}}} \right) \, \;{\text{for}}\;{\text{strike - slip}}\;{\text{regimes }}\left( {{\text{SS}}} \right) \\ {\text{R}}\prime & = \, \left( {{2} + {\text{R}}} \right)\;{\text{ for}}\;{\text{thrust}}\;{\text{faulting}}\;{\text{regimes }}\left( {{\text{TF}}} \right) \\ \end{aligned}$$

In this study, we derived the stress from the focal mechanism solutions database for the earthquakes which occurred in the vicinity of the Gulf of Aqaba. These earthquakes are listed in Table 4, and their focal mechanism parameters are listed in Table 5. The stress tensor inversion was derived for each basin in the Gulf, including the Dakar basin in the south, the Aragonese basin in the central, and finally the Eilat basin in the northern part of the Gulf. The results of stress inversion are evaluated according to the quality ranking starting from A to D. This quality was updated using the quality ranking system of the World Stress Map Release 2008^6^ where:

- A—quality: (SHmax/SHmin ± 15°): N ≥ 15 and α ≤ 12°.
- B—quality: (SHmax/SHmin ± 15°–20°): 8 < N < 15 and 12° pr < α ≤ 20°.
- C—quality: (SHmax/SHmin ± 20°–25°): 6 ≤ N < 8 or α > 20°.
- D—quality: (SHmax/SHmin ± 25°–40°) for the boxes with only 4 or 5 events.

**Table 4:** The hypocentral parameters for all earthquakes used in the construction of the fault plane solutions in the Gulf of Aqaba. (Example from EV01 to EV 37).

Event_no	Date			Time			Location		Depth (Km)	Mag
	Year	Month	Day	Hour	Min	Sec	Lat	Long		
EV01	1982	3	23	10	48	0	27.90	34.30	10	4.7
EV02	1985	12	31	19	42	41	29.13	34.90	9	4.8
EV03	1988	7	8	11	11	7.52	29.07	34.80	12	2.5
EV04	1988	8	20	9	57	23.92	29.09	34.79	15	2.6
EV05	1988	8	31	10	17	29.69	28.44	34.41	13	3.8
EV06	1988	9	2	19	25	21.52	29.08	34.78	18	3.0
EV07	1988	9	2	19	25	21.52	29.08	34.78	18	3.0
EV08	1988	11	9	21	6	57.36	28.79	34.72	12	3.2
EV09	1989	9	9	5	16	0	28.57	34.82	10	4.1
EV10	1990	7	2	12	11	44.74	28.63	34.24	89	3.4
EV11	1993	7	3	23	34	10	28.86	34.82	18	4.7
EV12	1993	8	3	12	43	5	28.73	34.55	17	6.0
EV13	1993	8	3	16	33	23	28.36	34.08	15	5.7
EV14	1993	8	6	6	51	26.62	28.74	34.52	12	4.2
EV15	1993	8	6	22	5	23.97	28.70	34.70	17	2.9
EV16	1993	8	7	4	55	40	28.61	34.63	10	4.2
EV17	1993	8	7	10	25	24.24	28.70	34.74	15	2.9
EV18	1993	8	9	6	5	4.33	28.75	34.68	12	4.6
EV19	1993	8	12	7	8	4.96	28.63	34.60	15	4.1
EV20	1993	8	12	19	0	43.49	28.67	34.67	12	4.2
EV21	1993	8	15	15	35	3.85	28.77	34.74	19	2.7
EV22	1993	8	16	11	14	39.77	28.70	34.61	11	3.7
EV23	1993	8	16	16	29	21.27	28.67	34.77	23	2.4
EV24	1993	8	20	23	9	59	28.72	34.61	2	4.6
EV25	1993	9	7	6	48	46.97	28.70	34.78	20	2.3
EV26	1993	9	13	0	13	54.41	28.66	34.52	4.5	3.7
EV27	1993	9	14	18	2	14.13	28.48	34.73	26	2.9
EV28	1993	9	15	2	46	52.71	28.65	34.72	15	2.0
EV29	1993	9	16	5	41	38.49	28.65	34.72	23	2.8
EV30	1993	9	20	1	34	15.53	28.69	34.76	21	2.5
EV31	1993	9	20	20	18	2.35	28.66	34.60	8.6	4.3
EV32	1993	9	25	12	38	10.06	28.63	34.78	20	2.8
EV33	1993	9	25	23	57	3.85	28.65	34.75	19	2.7
EV34	1993	9	26	16	25	10.42	28.71	34.66	17.5	2.7
EV35	1993	9	26	23	19	2.83	28.64	34.71	20	2.6
EV36	1993	11	3	18	39	32	28.70	34.65	7	4.9
EV37	1993	11	8	1	6	2	28.69	34.65	8	4.7

**Table 5:** The focal mechanism parameters for earthquakes in the Gulf of Aqaba source zone. (Example from EV01 to EV 37).

Index	Mechanism			P -axis		T-axis		Type	Reference
	Strike	Dip	Rake	az	pl	az	pl		
EV01	220	65	− 40	180	45	277	6	NS	Salamon et al.^72^
EV02	169	64	− 147	28	41	295	2	NS	Hofstetter et al.^23^
EV03	188	64	− 65	138	63	260	15	SS	Al-Arifi^73^
EV04	179	19	− 270	89	26	269	64	SS	–
EV05	76	70	− 100	331	64	174	25	NF	–
EV06	14	47	− 100	213	83	111	2	NF	–
EV07	14	47	− 100	213	83	111	2	SS	–
EV08	73	59	− 104	308	72	173	13	NF	–
EV09	205	50	− 110	50	74	309	3	NF	Salamon et al.^72^
EV10	42	77	− 72	335	55	117	29	NS	Al-Arifi^73^
EV11	84	72	− 151	306	33	212	6	SS	Abou Elenean^11^
EV12	357	61	− 68	309	67	72	13	NF	CMT Harvard solution
EV13	356	79	− 83	275	55	80	34	UF	–
EV14	8	28	− 153	193	51	324	28	NS	Al-Arifi^73^
EV15	78	87	− 77	2	47	156	40	NF	–
EV16	348	59	− 16	311	32	214	11	SS	Abou Elenean^11^
EV17	24	42	− 81	47	83	287	3	UF	Al-Arifi^73^
EV18	63	37	− 147	255	53	12	19	NS	–
EV19	44	46	− 80	34	82	127	0	NF	–
EV20	103	69	− 117	336	57	213	20	NF	–
EV21	34	59	− 67	350	67	108	11	SS	–
EV22	121	65	− 94	24	70	214	20	NF	–
EV23	34	48	− 62	17	70	285	1	NF	–
EV24	338	61	− 11	300	28	203	13	SS	Abou Elenean^11^
EV25	41	61	− 77	340	71	122	15	NF	Al-Arifi^73^
EV26	61	88	− 86	335	47	148	42	UF	–
EV27	10	81	− 127	245	42	129	27	NF	–
EV28	21	21	− 90	111	66	291	24	NF	–
EV29	57	68	− 67	1	60	130	20	SS	–
EV30	57	71	− 67	358	58	130	23	NF	–
EV31	68	50	− 132	271	59	6	3	NF	–
EV32	49	57	− 66	9	68	122	9	NF	–
EV33	164	51	− 70	135	74	240	4	UF	–
EV34	78	87	− 77	2	47	156	40	NF	–
EV35	57	64	− 59	12	59	124	13	TF	–
EV36	325	69	− 47	280	47	25	13	NS	Abou Elenean^11^
EV37	350	60	− 26	314	38	220	5	NS	–

Before computing the stress tensor using the focal mechanism solutions, we first validated the solution's degree of homogeneity and similarity by calculating the Kagan angle^67^. Kagan angle is a measure of the differences between the orientations of two fault planes in two different focal mechanism solutions. It detects and evaluates the minimum rotation angle between two source mechanisms. The Kagan angle varies between 0° (for identical solutions and full agreement between the two solutions) and120° (for absolute inconsistency and total conflict), the Kagan angle below 60° indicates a good correspondence while above 60° means a mismatch^68^. According to^69^ the pairs of solutions with an angle below 20°–30° were regarded as being very similar, while a Kagan angle of 60° is still considered as matching. The solutions that displayed a Kagan angle greater than 60° are excluded from this study. The results of the Kagan angle for the solutions that were selected in the Dakar basin ranges between 16° and 47° the majority of angles in the thirties. Out of the total of 21 solutions in this zone, we excluded 5 of them. The Kagan angle in the Aragonese basin ranges from 14.9° to 36.5° for normal solutions, with the majority falling in the twenties, and from 36° to 47.9° for strike-slip solutions, with the most falling in the forties. There are 50 total solutions in this basin; however, 20 of them are excluded. In the Eilat basin, the Kagan angle ranges from 10.14° to 44° with the majority in the thirties Out of a total of 25 solutions, 10 are excluded.

## Results

### Results of focal mechanism solutions

**Figure 6:**
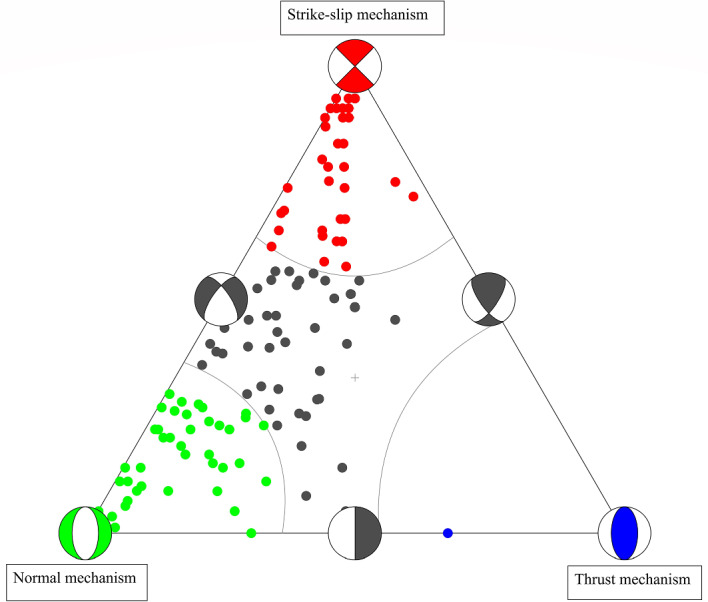
Ternary diagram, a graphical representation of focal mechanism orientations. The vertices of the triangle represent mechanisms with vertical T (thrust mechanisms), P (normal), and B axes (strike-slip).

**Figure 7:**
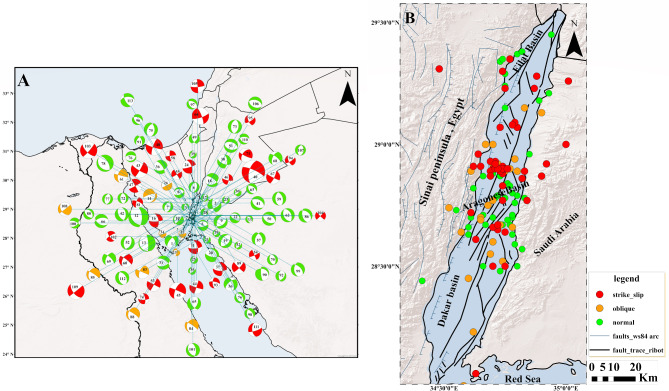
(**A**) Shows the beachball diagram for earthquakes that happened in the Gulf of Aqaba during the period from 1982 to 2021. (**B**) Represent the hypocentral distribution of these earthquakes. These maps were created by ArcGIS 10.3 software.

The results of 113 focal mechanism solutions show diversity in solutions where some of the solutions give pure normal faults, some solutions give left-lateral strike-slip faults and the other solutions give oblique normal faults. This is clear in the ternary diagram given in Fig. 6 which reflects the complexity of the geological and tectonic setting of the Gulf. These solutions clarified that the nodal planes follow the NNW–SSE, NW–SE, and NNE–SSW trends with different dip directions as shown in (Fig. S3) (Supplementary Material) (only some of these planes represent the actual trends for faults in the Gulf of Aqaba so we decided to calculate the stress tensor inversion to detect the actual trends in the Gulf). The beachball diagrams of 113 focal mechanism solutions in the Gulf of Aqaba are shown in Fig. 7A, and their hypocentral information and focal mechanism parameters are listed in Tables 4 and 5. Figure 7B shows the epicentres distributions for the available solutions in the Gulf. This distribution demonstrates the presence of the normal faulting mechanism throughout the Gulf. If we look into each basin individually, for instance, the Eilat basin, we will see that the Normal and the Strike-slip faulting mechanisms are somewhat equal. In contrast, the Aragonese basin contains three different types of mechanisms, but the Normal faulting and oblique normal faulting mechanisms predominate while the Dakar basin exhibits both oblique normal and normal faulting mechanisms. According to calculations of the total seismic moment and energy released by each type of mechanism for each basin and the entire Gulf, the seismic moment and energy produced by the strike-slip mechanism were higher than those released as a result of the normal mechanism.

### The results of stress tensor inversion in the Gulf of Aqaba

**Figure 8:**
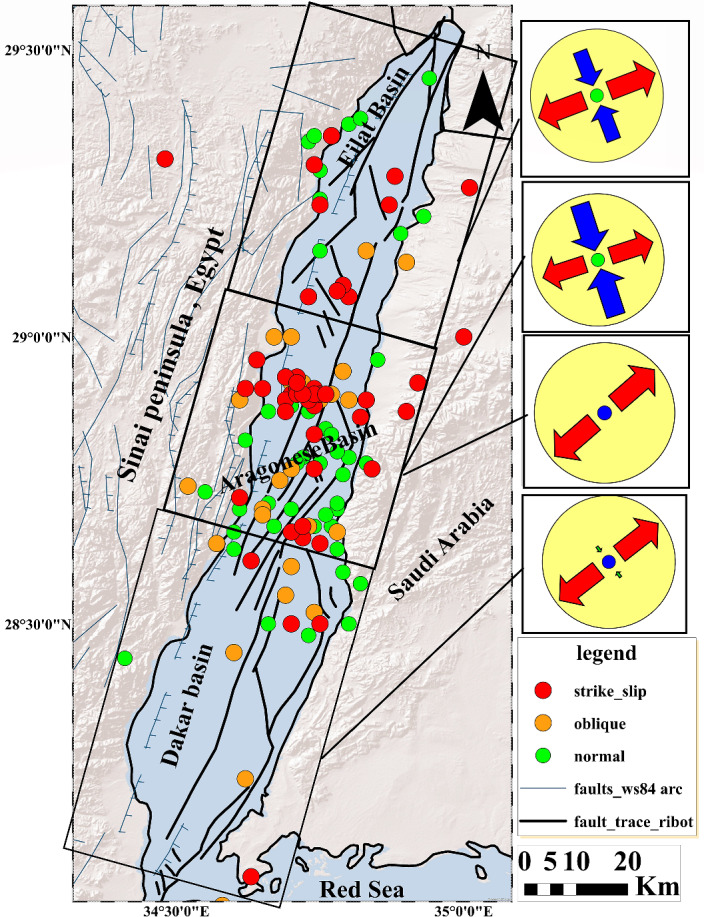
Stress tensor results for different zones in the Gulf of Aqaba. This map was created by ArcGIS 10.3 software.

The inversion technique described above was applied to a data set of 40 focal mechanism solutions in the Gulf of Aqaba. These solutions were subdivided into three different tectonic sub-regions including Dakar, Aragonese, and Eilat basins (Fig. 8). The inversion results of each sub-region will be discussed as follows:

#### Dakar basin stress tensor

**Figure 9:**
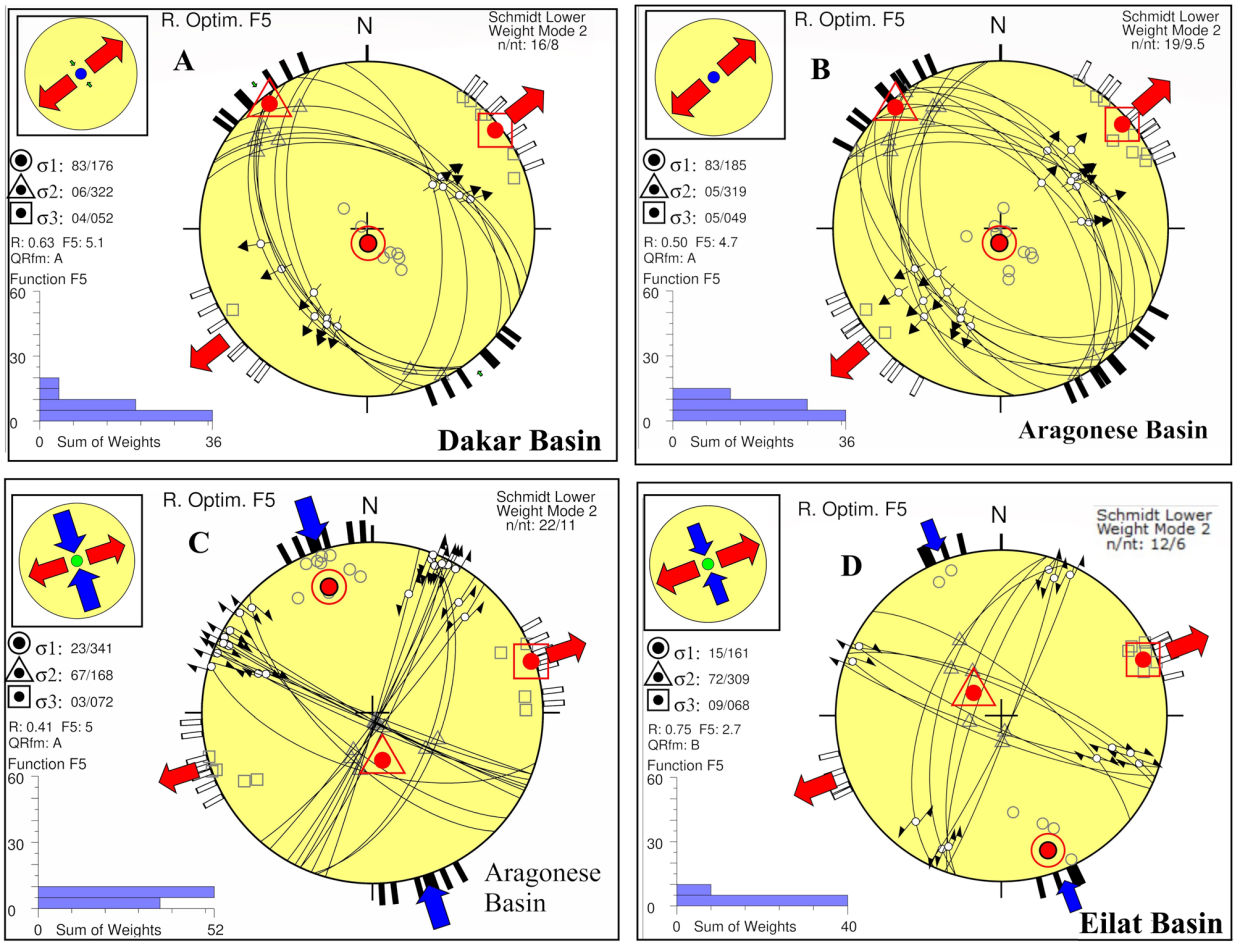
Stress tensor results in the Gulf of Aqaba. (**A**) Dakar basin, (**B**) normal stress regime in the Aragonese basin, (**C**) strike-slip regime in the Aragonese basin, and (**D**) Eilat basin.

The best-fitted stress tensor model obtained from a subset of 8 focal mechanism solutions in the Dakar basin (Fig. 9A) shows sub-vertical σ_1_ with a plunge of 83, sub-horizontal σ_3_ with a plunge of 4, and R′ = R which indicates that the basin is characterized by the normal faulting regime. The direction of the minimum horizontal stress SHmin is ENE (N52°E). The quality of the resulting stress tensor is A, with a low misfit angle (**α**) of **10.9** and an average misfit function (F5) of **5.1** as shown in Table 6.

**Table 6:** The parameters of stress inversion derived from focal mechanism solutions for the earthquakes that happened in the vicinity of the Gulf of Aqaba (divided as basins).

σ_1_		σ_2_		σ_3_		R	α	R^`^	F_5_	quality	SH_max_	Sh_min_	Stress regime
Az	Pl	Az	Pl	Az	Pl								
Dakar basin													
176	83	322	06	52	04	0.62	10.9	0.62	5.1	A	142	N52°E	NF
Aragonese basin													
185	83	319	05	49	05	0.50	10.1	0.50	4.7	A	139	N49°E	NF
341	23	168	67	72	03	0.41	11.5	1.59	5	A	162	N72°E	SS
Eilat basin													
161	15	309	72	68	09	0.75	13.2	1.25	2.7	B	159	N69°E	SS

#### Aragonese basin stress tensor

A reasonable examination of the focal mechanism parameters in the case of the Aragonese basin led to the separation of them into two dominating types. The first type involves 10 focal mechanism solutions. The best-fitting stress tensor model favours an extensional normal faulting regime with R′ of 0.5 which is supported by sub-vertical σ_1_ and subhorizontal σ_2_ axes with 83 and 05 plunges (Fig. 9B), respectively. The quality of the derived stress tensor is **A**, with a low misfit angle (**α**) of **10.1** and an average misfit function (F5) of **4.7** as shown in Table 6. The second type includes 11 focal mechanism solutions. The corresponding best-fitting stress tensor model indicates that the state of stress is dominated by horizontally to sub horizontally σ_1_ and σ_3_ Plunge 23 and 3, respectively while σ_2_ is close to vertical (Table 6, Fig. 9C). These findings manifest strike-slip regime with an **A** quality stress tensor, a low misfit angle (**α**) of **11.5** and an average misfit function (F5) is **5,** This regime is characterized distinguished by N72 E extensional direction and a stress regime R′ of 1.59.

#### Eilat basin stress tensor

The best-fitted stress tensor model obtained from a subset of 11 focal mechanism solutions in Eilat the basin indicates that the state of stress is dominated by horizontally to sub horizontally σ_1_ and σ_3_ Plunge 15 and 9, respectively while σ_2_ is close to vertical (72) as shown in Table 6 and Fig. 9D. These findings manifest a strike-slip regime with a **B**-quality stress tensor, a low misfit angle (**α**) of **13.5,** and an average misfit function (F5) of **2.7.** This regime is distinguished by N69 E extensional direction and a stress regime R′ of 1.25.

## Conclusions

The Gulf of Aqaba which occupies the southern end of the Dead Sea Transform Fault (DSTF) is the present-day most active tectonic zone in Egypt (as well as on the DSTF as a whole). This significant transform fault, which forms the plate boundary between Arabia and Africa—Sinai/Levant, is a North–South trending left-lateral strike-slip fault. During this study, we constructed fault mechanism solutions (FMS) for the earthquakes that occurred between 2012 and 2021 with local magnitude starting from 3.5 for updating and completing the database of fault plane solutions of earthquakes in the Gulf of Aqaba. These solutions clarified that the Gulf is characterized by the presence of different types of mechanisms such as normal faulting, strike-slip faulting, and oblique normal faulting. The database of the focal mechanism solutions has been separated into groups based on tectonic regionalization, including Eilat, Aragonese, and Dakar basins to evaluate the temporal changes of the stress field in these basins. The distribution of fault plane solutions demonstrated that normal faulting mainly affected the southern part of the Gulf (the Dakar basin), three different types of mechanisms affected the Aragonese basin, and a strike-slip mechanism affected the Eilat basin. The seismic moment and energy released by the strike-slip mechanism in the Gulf of Aqaba are greater than those released by the normal faulting mechanism, demonstrating the significant effect of the strike-slip movement along the Dead Sea Transform Fault.

By employing the inversion approach developed by^6^, which aims to select the actual nodal plane that most closely resembles the homogeneous stress field while calculating the stress tensor, it has been possible to determine the average stress field acting on each basin from the focal mechanisms of earthquakes from that basin. An additional analysis of the focal mechanism data that is currently available for the Aragonese basin, involving the separation of various stress states, has revealed the existence of a second stress state. The spatial variations of the stress field were analyzed based on the focal mechanism solutions with ML ≥ 2.5. The method developed by^67^ was applied to confirm the homogeneity of the focal mechanism data for each basin prior to the implementation of the inversion, and as we mentioned earlier, a Kagan angle of 60° is still regarded as matching. Kagan angle results showed that for the Eilat Aragonese and Dakar basins, respectively, 60% (the percentage of fault planes that satisfied the conditions of Kagan angle), 66.6%, and 76.19% of fault planes satisfied the requirements of Kagan angle within the thirties Degree.

The results of our analysis showed that the stress field in the Dakar basins is different from is not one would expect from the Dead Sea Transform Fault. The stress field in this basin displays an ENE-WSW extensional stress regime with the maximum compressional principal stress axis (σ1) being sub-vertical and the minimum extensional principal stress axis (σ3) being nearly horizontal. This stress pattern clearly illustrates how the Dakar basin has been affected by the incipient oceanic spreading in the northern Red Sea. The identified direction of extensional stress in the Dakar basin is nearly compatible with the direction of extension in the northern Red Sea. Our stress inversion for the Aragonese basin reveals two dominating stress patterns, a strike-slip regime, and an extensional regime. Subhorizontal orientation of both σ1 and σ3 with WNW and ENE trends, respectively are recognizable in the strike-slip regime. The second stress state is an extensional regime, which reflects ENE-WSW direction of extension. With a sub-vertical plunge of σ1 and a subhorizontal plunge of σ3. The orientations of σ3 in the Dakar and Aragonese basins are nearly similar to the direction of extension in the northern Red Sea, in conformity with the results of^14^ based on the fault slip direction data in the southern part (Dakar basin) of the Gulf. The existence of two stress regimes in the Aragonese basins reflects the interplay between the incipient spreading centre in the northern Red Sea and the strike-slip motion along the Aqaba Dead Sea Transform Fault. The best fitting solution for the Eilat basin showed a strike-slip regime with subhorizontal axes for both σ1 and σ3, with WNW and ENE trends, which fitted to the regional stress field along the Aqaba-Dead Sea transform fault.

A difficulty arises when comparing our results to those obtained from previous studies because our inversion is performed separately for three subsets of focal solutions that were categorized based on the tectonic regionalization of the Gulf of Aqaba, whereas previous studies inverted the solutions for the whole Gulf without any categorization^11,13,15,16^, or with a different categorization scheme^12^.

Finally, we may draw the following conclusion from studying FMS and stress tensor inversion in the Gulf of Aqaba: the tectonic setting in the Gulf of Aqaba is quite complex. Not just the DSTF's movement, but also the movement in the Red Sea, has had a significant impact on it. While the movement along the Dead Sea Transform Fault continues to have a significant impact on the Eilat basin, the Red Sea's influence extends totally to the Dakar basin and partially to Aragonese basin which is still significantly affected by the Dead Sea Transform Fault strike-slip movement. The seismic moment and energy released resulting from the strike-slip mechanism are larger than those released from the normal mechanism.

## Supplementary Information

Supplementary Figures.

## Data Availability

The datasets used during the current study are available from the corresponding author upon reasonable request.
