# Supplementary material for: Updating the seismotectonic setting for the Gulf of Aqaba
Source: Sci Rep. 2023 Jul 19;13:11672. doi: 10.1038/s41598-023-38759-6 (PMC10356856; doi:10.1038/s41598-023-38759-6)
Supplement: Supplementary file 1 — Supplementary Figures. [file 41598_2023_38759_MOESM1_ESM.docx]

***Supplementary material***

| 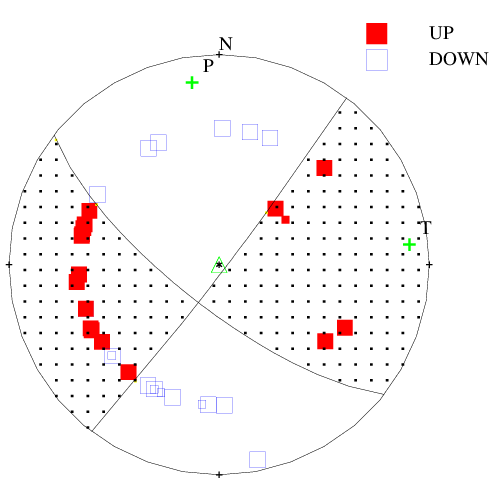Focal mechanism of Ev01 | 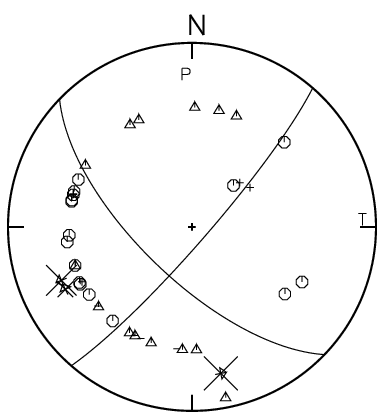Focal mechanism of Ev01 |
| --- | --- |
| 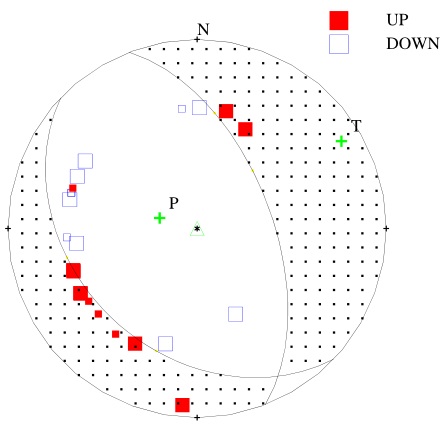Focal mechanism of Ev02 | 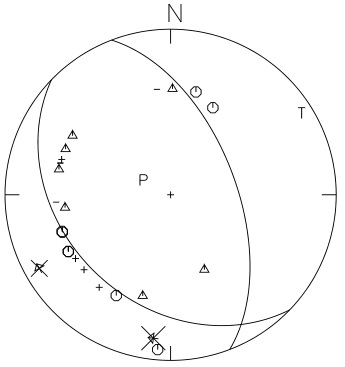  Focal mechanism of Ev02 |
| 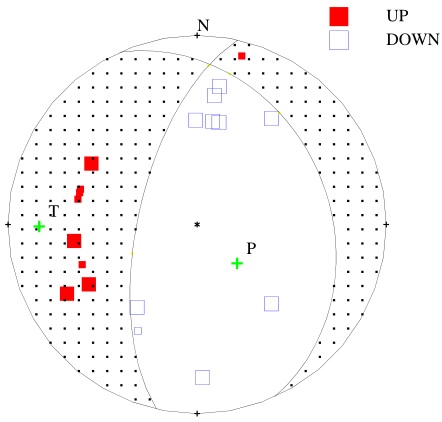Focal mechanism of Ev03 | 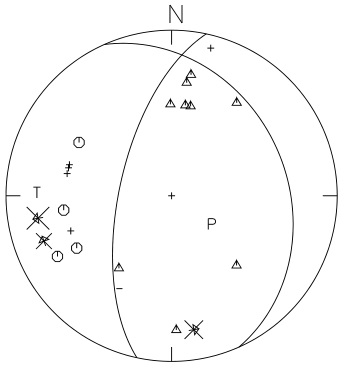  Focal mechanism of Ev03 |
| 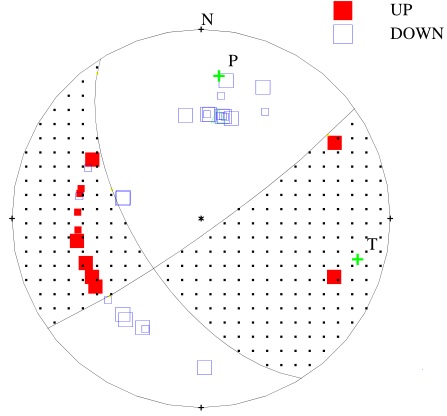Focal mechanism of Ev04 | 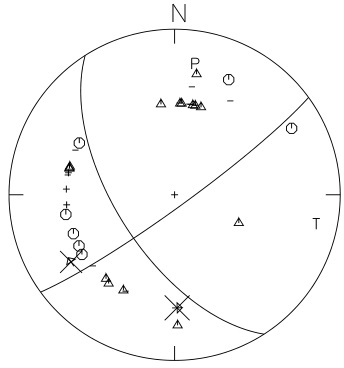Focal mechanism of Ev04 |
| 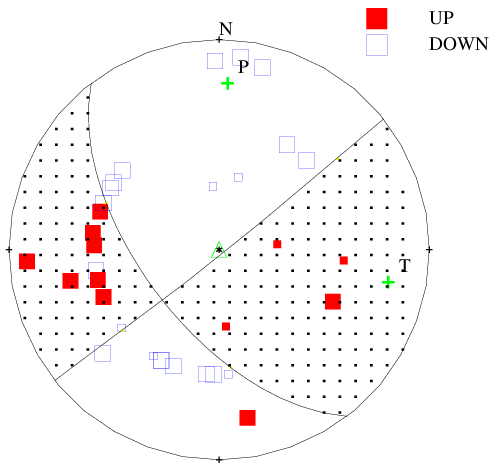Focal mechanism of Ev05 | 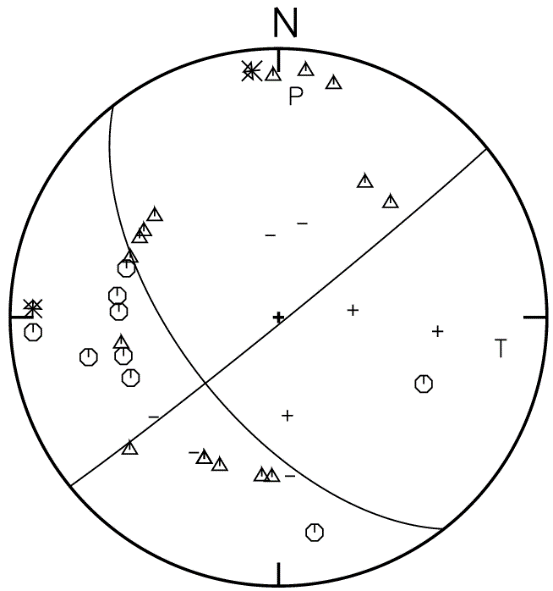Focal mechanism of Ev05 |
| Focal mechanism of Ev0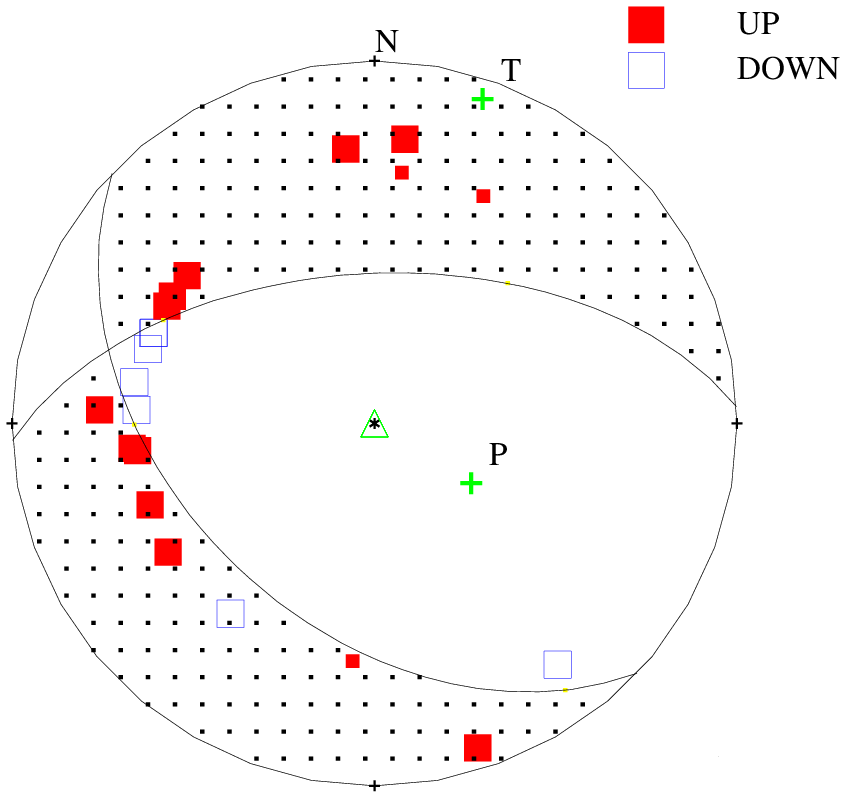6 | 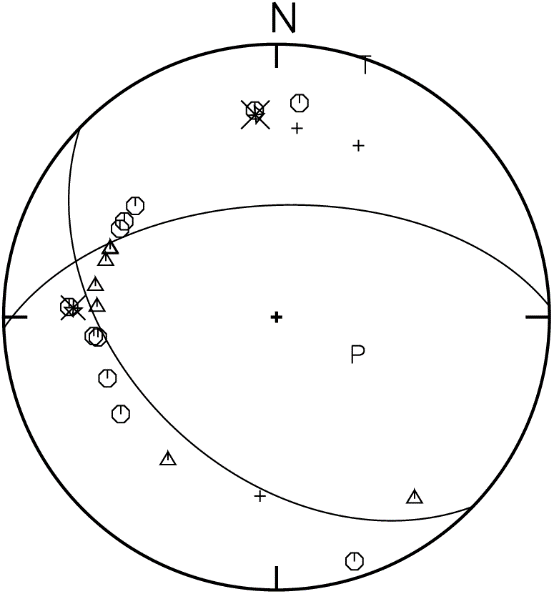Focal mechanism of Ev06 |
| Focal mechanism of Ev0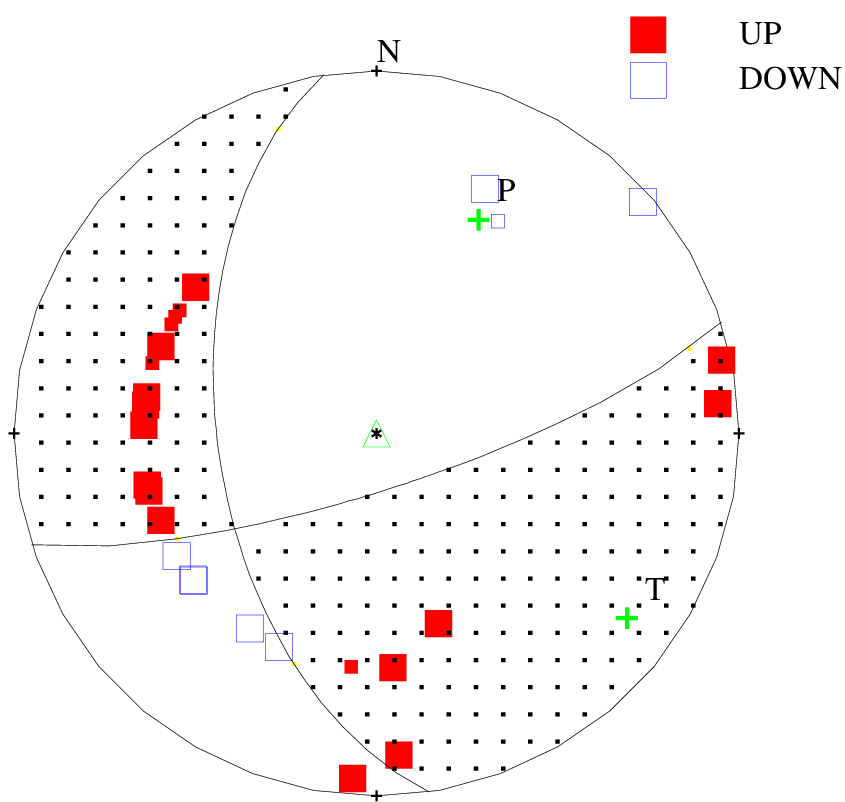7 | 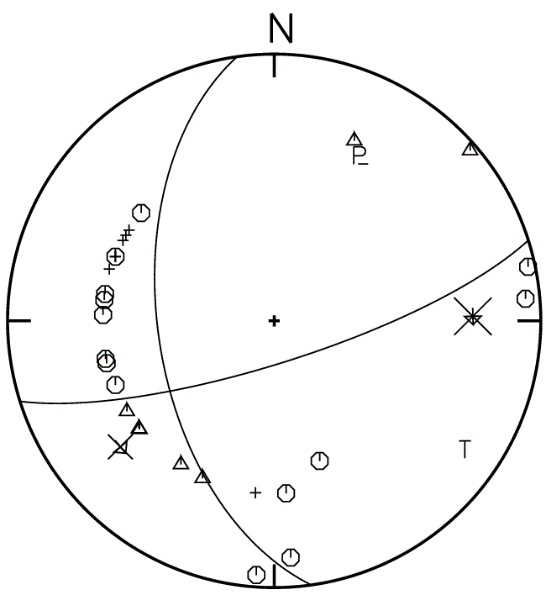Focal mechanism of Ev07 |
|  |  |

| 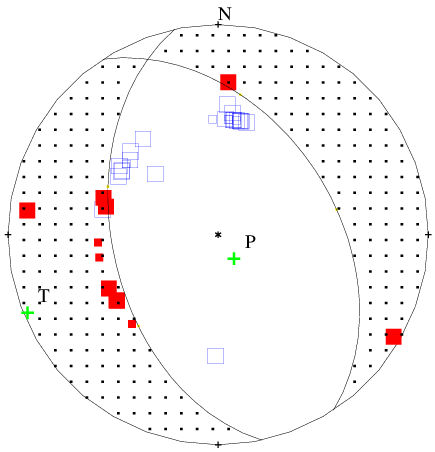  Focal mechanism of Ev08 | 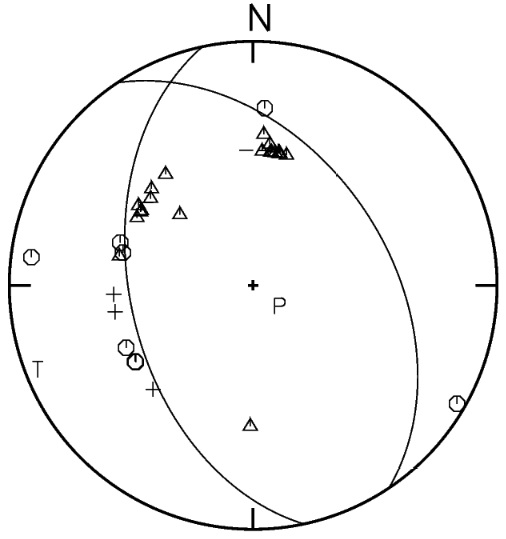Focal mechanism of Ev08 |
| --- | --- |
| Focal mechanism of Ev0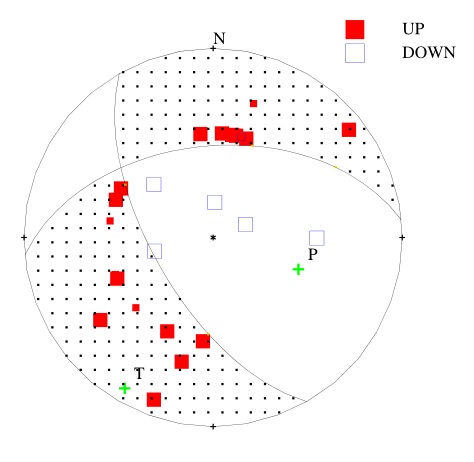9 | 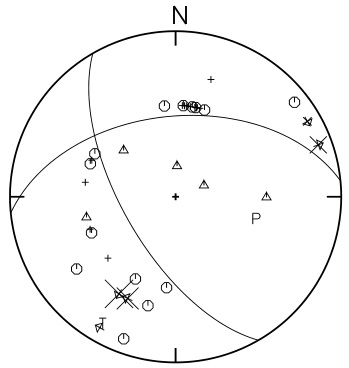  Focal mechanism of Ev09 |

Fig. S1: show the focal mechanism solutions for the earthquakes that happened in the vicinity of the Gulf of Aqaba between 2012 and 2021. The solutions on the left by Sutsugo and the right resulted by focmec software


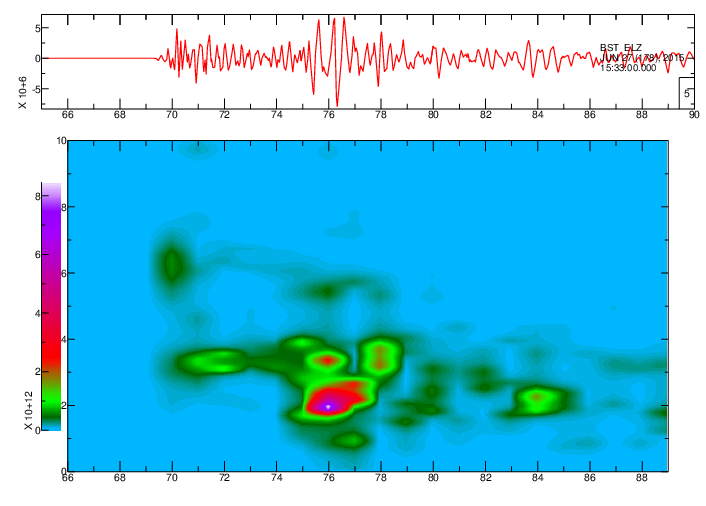

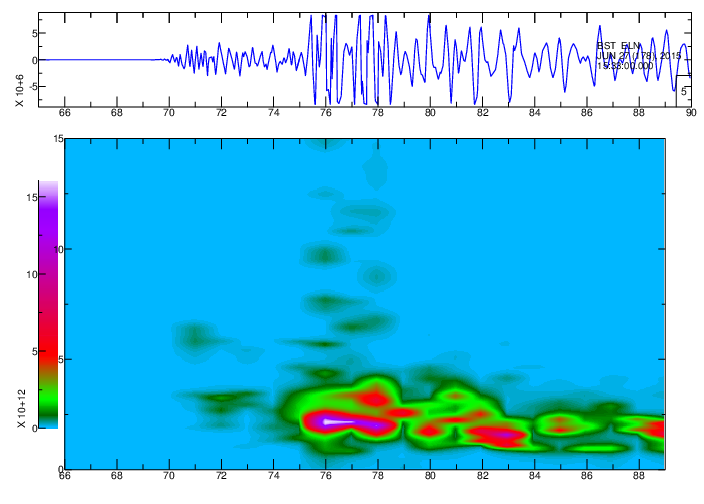

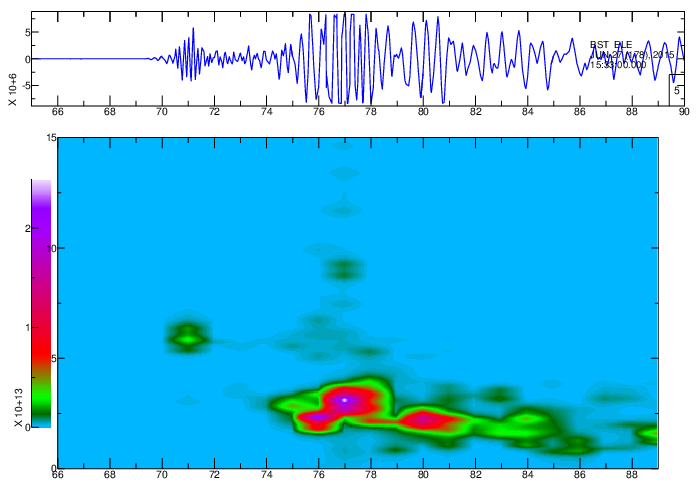


Fig S2: show the spectrogram for vertical and horizontal components to detect the duration of P-phase and S-phases.


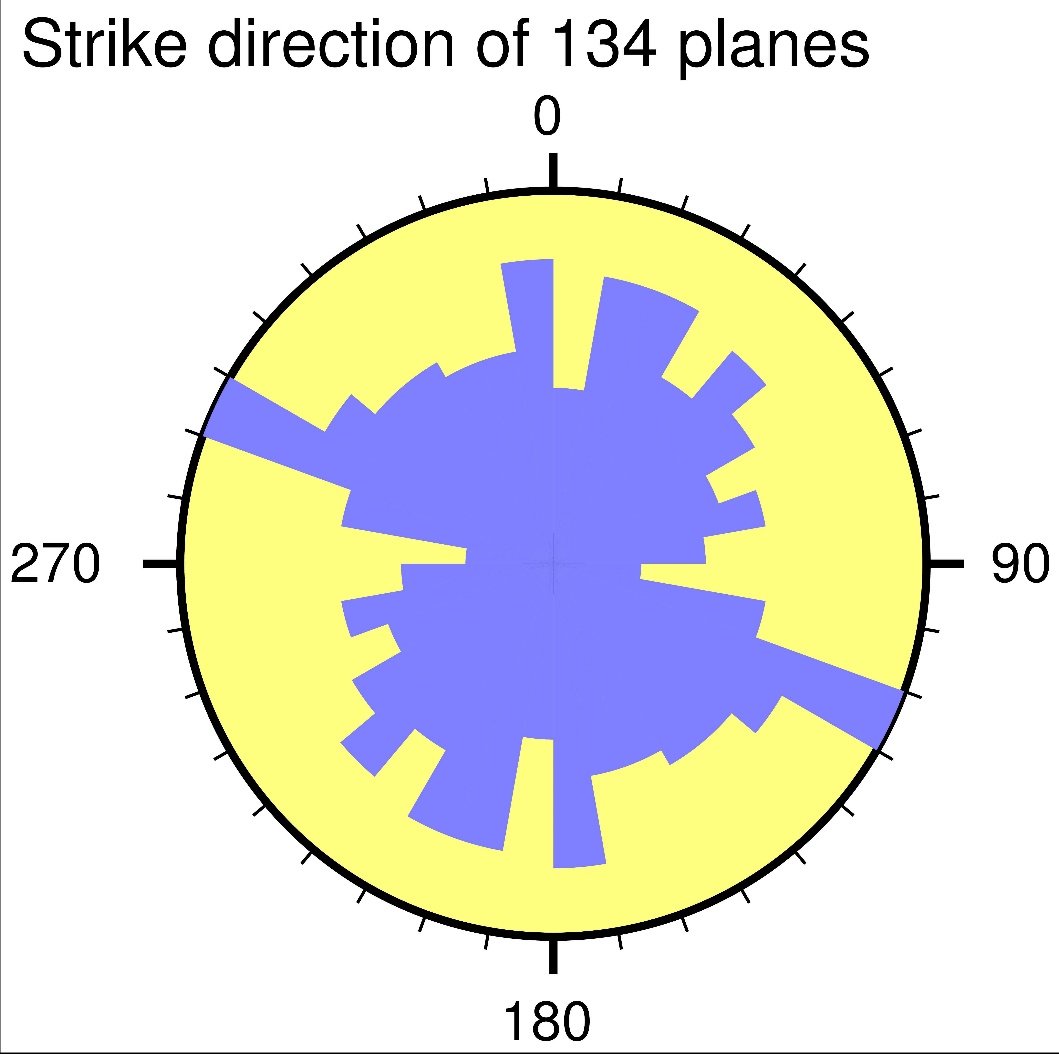


Fig.S3: Rose diagram shows the strike direction that represents trends of nodal planes for earthquakes in the Gulf of Aqaba (only some of these planes represent the actual trends for faults in the Gulf of Aqaba so we decided to calculate the stress tensor inversion to detect the actual trends in the Gulf).
